# RETRACTION: Circular RNA_0001946 is Insufficiently Expressed in Tumor Tissues, While its Higher Expression Correlates With Less Lymph Node Metastasis, Lower TNM Stage, and Improved Prognosis in NSCLC Patients

**DOI:** 10.1002/jcla.70360

**Published:** 2026-09-22

## Abstract

**RETRACTION**: M. Zhang, F. Wen, and K. Zhao, “Circular RNA_0001946 is Insufficiently Expressed in Tumor Tissues, While its Higher Expression Correlates With Less Lymph Node Metastasis, Lower TNM Stage, and Improved Prognosis in NSCLC Patients,” *Journal of Clinical and Laboratory Analysis* 35, no. 8 (2020): e23625, https://doi.org/10.1002/jcla.23625.

The above article, published online on 03 July 2021 in Wiley Online Library (wileyonlinelibrary.com), has been retracted by agreement between the journal Editor‐in‐Chief, Rong Fu; and Wiley Periodicals, LLC. The retraction has been agreed due to concerns raised by a third party that identified indications of compromised data and systematic issues within the published article, including inconsistencies related to patient recruitment. The authors did not provide the requested documentation relating to ethics approval, patient informed consent, or the underlying raw data. Accordingly, the article is retracted as the editors have lost trust in the reliability of the data and the results presented. The authors have been informed of this decision but were not available for final confirmation.

**RETRACTION**: M. Zhang, F. Wen, and K. Zhao, “Circular RNA_0001946 is Insufficiently Expressed in Tumor Tissues, While its Higher Expression Correlates With Less Lymph Node Metastasis, Lower TNM Stage, and Improved Prognosis in NSCLC Patients,” *Journal of Clinical and Laboratory Analysis* 35, no. 8 (2020): e23625, https://doi.org/10.1002/jcla.23625.

The above article, published online on 03 July 2021 in Wiley Online Library (wileyonlinelibrary.com), has been retracted by agreement between the journal Editor‐in‐Chief, Rong Fu; and Wiley Periodicals, LLC. The retraction has been agreed due to concerns raised by a third party that identified indications of compromised data and systematic issues within the published article, including inconsistencies related to patient recruitment. The authors did not provide the requested documentation relating to ethics approval, patient informed consent, or the underlying raw data. Accordingly, the article is retracted as the editors have lost trust in the reliability of the data and the results presented. The authors have been informed of this decision but were not available for final confirmation.

